# Endoscopic Perspective in Cholangiocarcinoma Diagnostic Process

**DOI:** 10.1155/2019/9704870

**Published:** 2019-12-20

**Authors:** O. Urban, P. Vanek, V. Zoundjiekpon, P. Falt

**Affiliations:** 2nd Department of Internal Medicine-Gastroenterology and Geriatrics, Faculty of Medicine and Dentistry, Palacký University Olomouc and University Hospital Olomouc, Czech Republic

## Abstract

Cholangiocarcinoma is a malignancy arising from the epithelial lining of the intrahepatic or extrahepatic biliary tract. Timely diagnosis is challenging due to its silent clinical course. As reliable laboratory markers are lacking, diagnostic imaging plays a pivotal role. While cross-sectional imaging studies are usually conclusive for intrahepatic lesions, endoscopy plays an essential role in cases of extrahepatic tumors. Rational utilization of different diagnostic methods based on available evidence is needed. This article focuses on the diagnostic role of advanced biliary endoscopy, including endoscopic retrograde cholangiopancreatography, cholangioscopy, endoscopic ultrasonography, and intraductal sonography.

## 1. Introduction

A timely diagnosis of cholangiocarcinoma remains challenging due to its silent clinical course. As reliable laboratory markers are lacking, diagnostic imaging plays a pivotal role. While cross-sectional imaging studies are usually conclusive for intrahepatic lesions, endoscopy plays an essential role in cases of extrahepatic tumors. Nevertheless, tissue specimen is usually necessary in both locations. This article addresses cholangiocarcinoma from the perspective of endoscopic imaging and tissue sampling.

## 2. Epidemiology

CCA is a malignancy arising from the epithelial lining of the intrahepatic or extrahepatic biliary tract, excluding the gallbladder, cystic duct, and ampulla of Vater. It accounts for 2% of all human malignancies and is the second most common primary hepatic malignancy following hepatocellular carcinoma.

The highest global incidence of CCA is in north-east Thailand where infestation by the liver fluke *Opisthorchis viverrini* is endemic. In this geographic area, age-standardized incidence rates of approximately 100 per 100,000 individuals among men and 50 per 100,000 individuals among women have been reported, while it ranges between 0.5 and 2.0 per 100,000 individuals in the Western world [1, 2].

As the incidence of CCA increases with age, the median age at diagnosis is 65-68 years. Sex- and race-specific disparities have also been noted. In the USA, males and specifically Asians are affected more frequently than females and non-Hispanic whites [3].

Worldwide time trends in mortality for CCA are changing. Multiple studies have reported that the incidence of ICCA has increased by up to tenfold in high-income countries, while the incidence of ECCA has decreased at a similar rate around the turn of the 20^th^ century. Nevertheless, it has also been suggested that this shift may have been influenced by implementing different classification systems [4, 5].

## 3. Prognosis and Treatment

The prognosis of biliary malignancies is dismal with the overall five-year survival as low as 10%. Radical surgery remains the only option for curative therapy offering a median disease-free survival of 12-36 months. However, due to the silent clinical course, two-thirds of CCAs are diagnosed at an inoperable stage. The natural course without chemotherapy or radiotherapy leads to overall survival of 3.9 months only. If palliative chemotherapy is used, median survival can be prolonged up to 12-15 months [6, 7]. After resection, negative margin (R0 resection) is the most important variable associated with long-term survival [8]. Recently, an excellent five-year survival of 65% in patients with “very early” perihilar cholangiocarcinoma was demonstrated after neoadjuvant chemoradiation followed by liver transplantation [9].

## 4. Etiopathogenesis

While the etiopathogenesis remains unknown, the development of biliary cancer is linked to a wide spectrum of conditions causing biliary inflammation, cholestasis, and inflammation of the liver. Different precancerous conditions and recommendations for the follow-up of patients with increased risk are described in detail elsewhere. Despite increasing knowledge within this field, the diagnosis of CCA at an early stage remains a challenge since the majority of cases are sporadic, affecting patients without the presence of any known risk factor [10].

## 5. Diagnostic Approach

The clinical presentation of CCA depends on tumor location. The typical triad of ECCA consists of symptoms of biliary obstruction, right upper quadrant pain, and weight loss. However, patients with jaundice have mostly advanced disease already. Therefore, making a diagnosis of ECCA in a nonjaundiced person remains a crucial issue. It has been shown by Sugiyama et al. that abnormalities in hepatic function and tumor marker tests combined with transabdominal ultrasonography (US) can be used for early diagnosis of ECCA in patients without jaundice, with resulting resectability of 83% and survival of 50% at 5 years [11].

A reliable laboratory test for CCA is still lacking. Extrahepatic cholestasis is reflected in elevated conjugated bilirubin, alkaline phosphatase, and gamma-glutamyltransferase levels. As for “tumor markers,” it is widely accepted that testing for the carbohydrate antigen (CA) 19-9 is useful in diagnostics, as a prognostic factor and indicator of tumor resectability. However, the levels of CA 19-9 can also increase in other hepatobiliary conditions, including cholangitis. On the other hand, about 4-14% of the population with Lewis-negative phenotype are not able to secrete CA 19-9 even when malignant tumor is present [12].

Due to the unspecific clinical presentation and the absence of accurate laboratory tests, the diagnosis of CCA depends mainly on imaging with or without tissue sampling. For ICCA, cross-sectional imaging methods are mostly conclusive. On the other hand, the role of advanced pancreatobiliary endoscopy is essential in the cases of ECCA, as will be discussed further in this text.

## 6. Classifications

Most classifications of CCA were developed to guide surgical and oncological treatment. Nonetheless, they can also be used for tailoring palliative treatment including endoscopic or percutaneous drainage and locoregional therapy.

As with most other tumors, CCA can be staged according to the tumor, node, metastasis (TNM) system [13]. Yet, the TNM system is unable to provide the information necessary for an assessment of surgical resectability in the case of ECCA. Therefore, additional staging systems based on anatomical, pathological, and surgical characteristics are also used [14].

**Figure 1:**
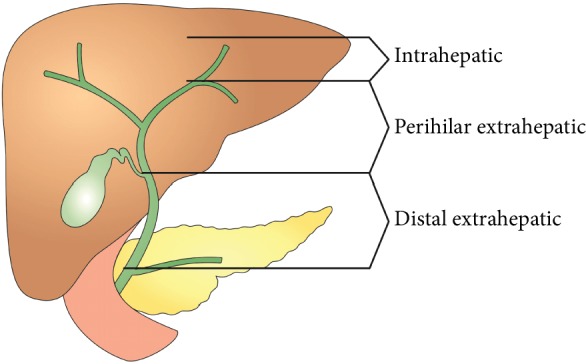
Classification of cholangiocarcinoma according to the anatomical location.

According to the anatomical location, CCA can be divided into ICCA and ECCA with the anatomical border at the level of second-order bile ducts. As for ECCA, it can be subdivided into PCCA and DCCA, two entities anatomically separated by the insertion of the cystic duct into the common bile duct. From all CCAs, the proportion of PCCA, DCCA, and ICCA is 50-60%, 20%, and 20%, respectively [15] (Figure 1).

**Figure 2:**
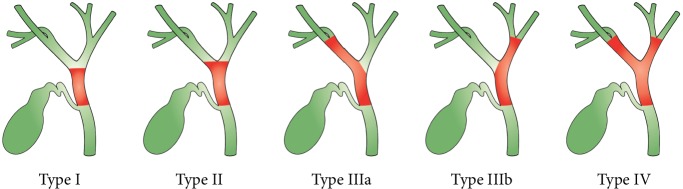
Classification of perihilar carcinoma according to Bismuth and Corlette.

PCCA, usually referred to as a Klatskin tumor, can be further described according to the *Bismuth and Corlette* classification as types I-IV: type I involves the common hepatic duct below the confluence of the right and left hepatic ducts, type II involves the confluence, type IIIa involves the confluence while extending into the right hepatic duct, type IIIb involves the confluence while extending into the left hepatic duct, and type IV involves the confluence while extending into both the right and left hepatic ducts, or it is multifocal [16, 17] (Figure 2).

**Figure 3:**
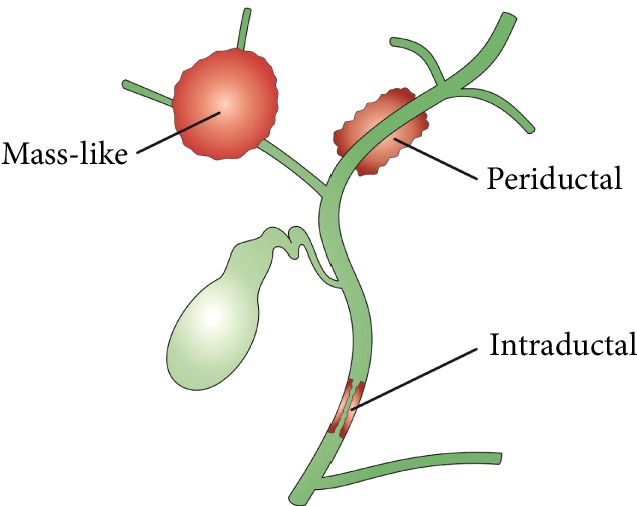
Classification of cholangiocarcinoma carcinoma according to the macroscopic morphology.

As for macroscopic morphology, three types of CCA have been described by the Liver Cancer Study Group of Japan including (1) periductal-infiltrating, (2) intraductal-papillary, and (3) exophytic or mass-forming types. The American Joint Committee for Cancer also recognizes mixed periductal-infiltrating and intraductal-growing types. The periductal infiltrative type is the most common, representing 70% of cases [18] (Figure 3).

It has been shown that the type of tumor spread correlates with the morphology growth pattern. While mucosal extension is predominantly seen with intraductal-papillary and mass-forming (nodular) tumors, submucosal extension is mainly seen with periductal-infiltrating types. Generally, the extension occurs more frequently to the hepatic side, where it also tends to reach further [19, 20].

Histologically, 90% of CCAs are adenocarcinomas that are well, moderately, or poorly differentiated. They consist of either cylindrical mucin-producing glands or cuboidal non-mucin-producing cells. All types of CCAs are associated with the rapid proliferation of tumor-associated stroma cells that contribute to desmoplasia.

Recently, a new staging system integrating important anatomic, pathologic, and surgical features of PCCA has been suggested by a group of international experts. Attempting to reflect a unique complexity of this tumor, it employs eight characteristics for staging: tumor extent, tumor size, tumor growth type, vascular involvement, hepatic lobar atrophy, underlying hepatic disease, lymph node metastases, and distant metastases [21].

## 7. Cross-Sectional Imaging

Cross-sectional imaging involves any technique that produces an image in the form of a plane through the body with the structures cut across.

### 7.1. Transabdominal Ultrasonography

Transabdominal ultrasonography (US) is frequently used as a first-line imaging test in jaundiced patients. While ICCA presents as a hypoechoic mass, the direct visualization of ECCA on US is usually impossible. In one study, the sensitivities of US in demonstrating hilar tumor, middle bile duct tumor, and distal bile duct tumor were 86%, 59%, and 33%, respectively [22]. US has nevertheless proven to be useful in detecting biliary tract dilation, level of obstruction, and the presence of gallstones.

### 7.2. Computed Tomography/Magnetic Resonance Imaging (PET/CT)

Computed tomography (CT) is performed in the vast majority of CCAs. This is not the place to discuss the role of CT in the diagnosis of ICCA. The performance of a CT scan as a diagnostic tool for PCCA was evaluated in a meta-analysis of 16 studies demonstrating an accuracy of 86% for the ductal extent of the tumor. Sensitivity and specificity were 89% and 92% for the evaluation of portal vein involvement, 83% and 93% for hepatic artery involvement, and 61% and 88% for lymph node involvement, respectively [23]. As for the PCCA resectability assessment, another meta-analysis calculated the pooled sensitivities of CT, MRI, and PET/CT to be 95%, 94%, and 91%, respectively, with corresponding specificities of 69%, 71%, and 81%. As concluded by the authors, CT is the most frequently used modality. The diagnostic performance of MRI proved to be generally comparable with CT, while PET/CT appears to be the best method in detecting lymph node and distant metastasis [24].

## 8. The Role of Endoscopy

### 8.1. Endoscopic Retrograde Cholangiopancreatography (ERCP)

Since its introduction in 1968, ERCP has been widely used for imaging of the biliary tree and is still considered the gold standard of biliary imaging. Nevertheless, ERCP remains an invasive procedure with considerable risks. As reported by Cotton et al., unavoidable overall, severe, and lethal complication rates of 4%, 0.7%, and 0.06%, respectively, must be expected even in the hands of experts [25].

The most important ERCP-related complication is pancreatitis occurring in 2.6% of patients followed by ascending cholangitis. Although several preventive measures have proven effective in post-ERCP pancreatitis prophylaxis, including the administration of certain drugs, guidewire cannulation technique, vigorous intravenous hydration, and pancreatic duct stenting, the exclusion of patients in whom ERCP is unnecessary remains a crucial point [26].

In expert centers, ERCP has been replaced by less-invasive diagnostic modalities such as CT, MRCP, and EUS at the position of the first-line imaging method. It has been shown that MRCP in combination with MRI has a comparable diagnostic accuracy with direct (ERCP or percutaneous transhepatic) cholangiography combined with CT in the detection and staging of ECCA [27]. However, ERCP is still being widely utilized for transpapillary tissue sampling. Moreover, the availability of MRI and EUS is not universal, and the position of ERCP in the diagnostic algorithm for ECCA may, therefore, vary according to the level of local expertise [28].

In ERCP, CCA presents as a stricture or a filling defect with or without upstream dilation of the biliary tract. Malignant etiology is suggested in a long (≥10 mm), asymmetric, and irregular stricture opposed to a short, regular, and symmetric stricture, which is typical for benign disease. As shown by Park et al., using these criteria, the diagnostic sensitivity and specificity for ERCP were 74% and 70%, respectively [29].

When necessary, material for histopathology examination can be obtained by one of 3 different approaches during ERCP: (1) bile aspiration, (2) brush cytology, and (3) endobiliary biopsy. While the specificity of diagnosing malignancy approaches 100% uniformly for each method, sensitivity varies widely and generally remains unsatisfactorily low. For instance, the sensitivity of cytology examination of aspirated bile ranges between 6 and 24% [30, 31].

Endoscopic brush cytology may be considered a standard method that is safe and easy to perform. Its diagnostic performance has been evaluated in many studies with the sensitivity for CCA in the range of 23-80%. In a meta-analysis of 16 studies including 1556 patients, Burnett et al. calculated its sensitivity as 42%. Two to five brush passes through the stricture have been suggested in various studies [28, 32].

**Figure 4:**
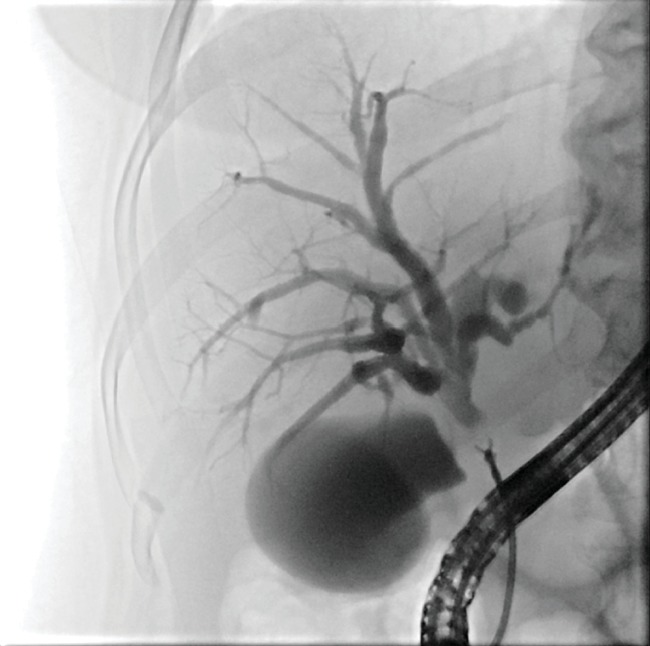
Endoscopic retrograde biopsy of biliary stricture.

As for endoluminal forceps biopsy, the sensitivity for CCA is in the range of 52-81%. Compared to brushing, forceps biopsy is technically more demanding and may require sphincterotomy. Biopsy can also be difficult or impossible in a narrow duct. The number of biopsy bites in studies ranges between 1 and 6. As shown by Tamada et al., three biopsy bites are sufficient to obtain sensitivity of 100% in patients with the papillary type CCA, while multiple biopsies are necessary for the infiltrating type [33–37] (Figure 4).

It makes sense that different sampling methods could be combined. In a meta-analysis of 9 studies, Navaneethan et al. found the pooled sensitivity of endoscopic brush cytology, intraductal biopsy, and combination of both to be 45%, 48%, and 59%, respectively, with corresponding specificity of 99%, 99%, and 100%. The authors concluded that both brushing and biopsy are comparable with limited sensitivity, which can only modestly be increased by the combination of both [38].

Several factors contribute to frequent false-negative results of CCA tissue analysis. Among them, general difficulties in tissue sampling, the desmoplastic paucicellular character of CCA, and background inflammatory changes are the most prevalent. In a study devoted to endoscopic brush cytology, Logrono et al. proved that tissue sampling error was the cause of false-negative findings in 67%, followed by technical error in 17% and interpretive error in the remaining 17% [39].

Different technical solutions have been investigated in order to increase successful tissue sampling. Among them, using a larger cytology brush (3 mm × 5 cm) increased cellularity but did not improve the cancer detection rate when compared to the standard brush. The combination of biliary dilation, endoscopic needle aspiration, and subsequent brushing cytology showed higher sensitivity (85%) than brushing alone (57%). A cytological evaluation of postbrushing biliary lavage fluid increased sensitivity by 24%. A dedicated basket for tissue grasp provided better sensitivity than brushing (80 vs. 40%). Moreover, combining brush cytology with CA 19-9 assessment brought better diagnostic accuracy in patients with primary sclerosing cholangitis [40–44].

To improve the sensitivity of routine cytology, advanced cytological methods can be used. Among them, fluorescence in situ hybridization (FISH) is a test employing fluorescently labelled DNA probes to detect gains or losses of chromosomal regions. As shown by Fritcher et al., a combination of FISH probes 1q21, 7p12, 8q24, and 9p21 identified pancreatobiliary malignancies with sensitivity of 93% and specificity of 100%. The typical finding was polysomy, indicating a presence of five or more cells with gains detected for two or more probes. In other studies, FISH proved to increase sensitivity from 20% to 43% as compared to routine cytology. In cases with negative brush cytology and forceps biopsy, FISH could predict malignancy in 62% of patients with an indeterminate biliary stricture [45, 46]. Next-generation DNA sequencing (NGS), another ancillary cytological method, has also turned out to be promising. In a study combining NGS with cytology, NGS increased sensitivity to 85% as compared to 67% for cytology alone [47]. Triple modality testing combining brush cytology, FISH, and biopsy showed overall sensitivity of 82% and specificity 100% [48].

To summarize the above-mentioned, while positive ERCP-guided tissue sampling can be regarded as diagnostically conclusive, a negative finding should be interpreted with caution. The term “indeterminate stricture” is used to indicate biliary strictures remaining of a likely malignant etiology despite negative pathology results. In such cases, surgical exploration must be considered in order not to miss the opportunity of curative surgery for a potential malignancy. With that being said, 5-30% of surgically treated lesions were reported benign on final histopathology according to different studies [49]. Currently, novel endoscopic modalities, including cholangioscopy, can be used to avoid unnecessary surgery.

### 8.2. Cholangioscopy

The history of cholangioscopy dates back to the 1970s when two different endoscopic approaches to the biliary tract were described: (1) the “mother-baby” technique using a small endoscope that was passed through the working channel of a duodenoscope and (2) peroral direct cholangioscopy using a pediatric or dedicated forward viewing endoscope with an additional bending function that were advanced through the mouth [50–52].

As shown mainly by Japanese endoscopists, cholangioscopy increases sensitivity to 96-100% for the diagnosis of malignancy when combined with ERCP [53–56]. Nonetheless, both these techniques had important limitations that prevented their widespread usage. The former “mother-baby” endoscopy required two operators and the “babyscope” was fragile. The peroral cholangioscope was difficult to manipulate, especially within nondilated ducts, plus the dedicated video cholangioscope did not become commercially available in the West [57–61].

The current technological standard for the “mother-baby” approach is represented by a digital single-operator cholangioscope (DSOC, SpyGlass DS; Boston Scientific, Inc., Natick, Massachusetts, USA) commercially available since 2015. SpyGlass comprises a disposable 10.5 Fr scope with an integrated digital sensor and portable processor. The scope allows four-way tip deflection and enables suction, irrigation, and passage of miniaturized biopsy forceps (SpyBite; Boston Scientific, Inc.) or any of several other accessories [62].

**Figure 5:**
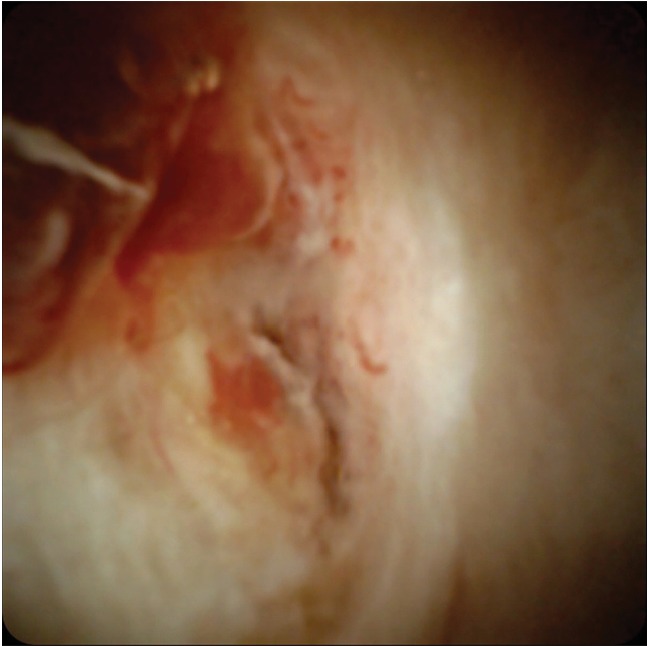
Cholangioscopy (SpyGlass) view of the intraductal-type cholangiocarcinoma.

**Figure 6:**
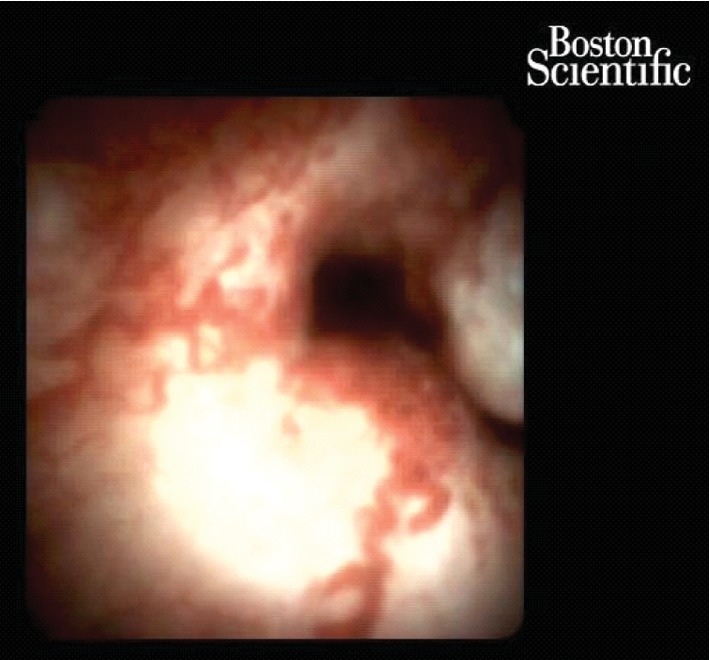
Cholangioscopy (SpyGlass) view of the periductal-type cholangiocarcinoma.

DSOC enables direct visualization of the biliary tract and intraluminal biopsy. Although no formal consensus on visual diagnostic criteria has been established, the presence of either nodular or papillary masses, irregular surface, tortuous dilated vessels, and fragile mucosa are considered features typical of neoplasia. For benign lesions, a flat surface, fine network of vessels, regular granular appearance, nonfragile mucosa, convergence of folds, and presence of pseudodiverticula are deemed diagnostic. The sensitivity and specificity of visual diagnosis were reported in the range of 90-100% and 76-96%, respectively [63–67] (Figures 5 and 6).

As for SpyBite biopsies, a sensitivity of 85-86% and specificity of 100% can be expected. Moreover, as shown by Varadarajulu et al., biopsy sensitivity can be increased up to 94% by using rapid on-site examinations of touch imprint cytology [68].

It has been suggested by Sugiyama et al. that the evaluation of ECCA extension may influence surgical strategy. Yet, as shown by Itoi et al., the proximal tumor margin can be visualized only in a limited number of patients and submucosal tumor extension is not possible to estimate at all. In one study comparing DSOC and ERCP, DSOC provided no additional information regarding the local spread of the disease in three surgically treated cases. Among these cases, an intraoperative frozen section revealed a spread of malignant cells beyond the predicted tumor margin in one of them, while the remaining two corresponded in preoperative and intraoperative findings. Thus, the results of local staging with DSOC should be interpreted cautiously [67, 69, 70].

As for the complications of the method, a retrospective study comparing ERCP with and without cholangioscopy showed increased morbidity when cholangioscopy was performed. Of note, the risk of cholangitis increased five times [71]. It was suggested that the higher risk of cholangitis is caused mainly by an increase in intraductal pressure due to intermittent water irrigation during the procedure. In a recent meta-analysis of 49 studies including 2193 patients who underwent either diagnostic or therapeutic peroral cholangioscopy, Korrapati et al. reported an overall and a serious adverse event rate of 7% and 1%, respectively. To prevent cholangitis, antibiotic prophylaxis and adequate biliary drainage are necessary [72].

Despite excellent operating characteristics, the position of DSOC in the diagnostic algorithm of uncertain biliary strictures must be further evaluated in regard to other modalities. Because of its costs, complexity, and procedure-related morbidity, DSOC should be considered mainly in the case of indeterminate biliary strictures in which previous ERCP tissue sampling was not conclusive. Even so, DSOC is becoming widely available and may become an option at hand during index ERCP in the future, particularly in cases with proximal strictures. Although such an approach could theoretically shorten the diagnostic process, its impact on safety remains to be investigated.

### 8.3. Endoscopic Ultrasound

**Figure 7:**
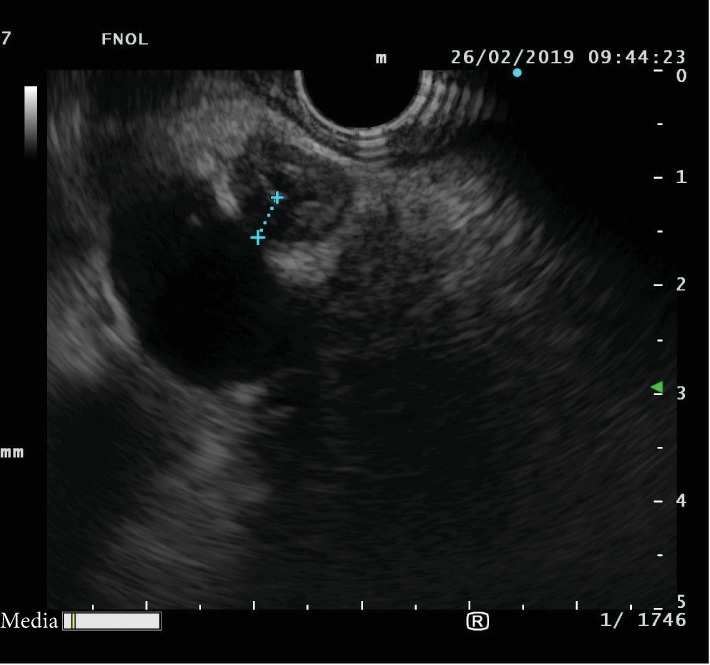
Linear endosonography view of a distal cholangiocarcinoma.

Endoscopic ultrasound (EUS) was introduced in clinical practice in the 1980s. Since then, the position of EUS in diagnosing and staging pancreatobiliary diseases, including CCA, has been firmly established. During the EUS procedure, CCA can be readily visualized in both cross-sectional and longitudinal views. If a mass is present, it usually appears hypoechoic or, less frequently, heterogenous (Figure 7). The upstream dilatation of the biliary tract can also be estimated. In addition to tumor depiction, EUS allows for the identification of the hilum, celiac axis, and para-aortic lymph nodes facilitating the staging process.

As for malignant biliary stricture detection, EUS without fine needle aspiration (FNA) was found to provide sensitivity of 78% and specificity of 84% [73]. Another study proved EUS to be superior in biliary cancer detection when compared to CT and MRI/MRCP (94%, 30%, and 42%, respectively), with a statistical significant difference. In the same study, EUS detected 100% of distal and 83% of proximal CCAs while demonstrating sensitivity of 53% and specificity of 97% in the assessment of unresectability [74].

The development of linear echoendoscopes enabled EUS-FNA which further improved the diagnostic capability of EUS. Across several studies, EUS-FNA sensitivity and specificity for differentiating ECCA from benign biliary lesions range between 43-89% and 79-100%, respectively. Moreover, sensitivity of 45% could be achieved even in the case of ECCA with no definite mass on cross-sectional imaging. Similar to EUS alone, the operating characteristics of EUS-FNA proved better for distal as compared to proximal lesions. One study has demonstrated sensitivity of 81% for distal and 59% for proximal lesions with the overall sensitivity being 73%.

Based on these results, EUS-FNA can be readily recommended for tissue sampling in cases with inconclusive results from ERCP brushing. A meta-analysis of patients with negative brush cytology has revealed the sensitivity and specificity of EUS-FNA to be 59% and 100%, respectively, for the diagnosis of ECCA [75–78].

The possibility of using EUS-FNA as a safer alternative to ERCP was also investigated. For instance, Onda et al. performed EUS-FNA as the first-line method in patients with suspected ECCA based on CT or other imaging modalities with sensitivity of 89% and accuracy of 87% [79]. In a recent meta-analysis, De Moura et al. calculated the mean sensitivities of ERCP and EUS-FNA for the tissue diagnosis of a malignant biliary stricture to be 49% and 75%, while specificities were 96% and 100% [80].

However, not all literature is in agreement with the superiority of EUS over ERCP in the assessment of malignant biliary strictures. For example, although the study by Rosch et al. proved EUS to be more sensitive in the diagnosis of pancreatic tumors, ERCP was a better diagnostic modality for CCA [75]. In a study by Weilert et al., ERCP brushing had the same sensitivity (79%) as compared to EUS-FNA [81].

Finally, same-session EUS-FNA and ERCP-based tissue sampling was superior to EUS-FNA alone for both pancreatic and biliary lesions [82].

It is important to note that the negative predictive values of EUS-FNA for malignancy were relatively low in most of the studies, ranging from 29% to 67%. Therefore, similarly to ERCP, a negative EUS-FNA may not exclude the malignant etiology of biliary strictures.

The short-term endoscopic risks of EUS-FNA are low, including acute pancreatitis (0.3-2%), bleeding (1%), perforation (0.4%), and infection (0.3%) [83].

Amid late complications, tumor cell seeding after transperitoneal FNA must be considered in operable patients. As reported by Heimbach et al., peritoneal metastasis occurred in 83% of patients who underwent a percutaneous or transluminal FNA biopsy of the primary hilar tumor mass as opposed to 8% in cases where no biopsy was performed [84]. As a result, the Mayo Clinic transplantation protocol excludes patients who have undergone a biopsy of the primary tumor from neoadjuvant therapy and liver transplantation. So far, there have been no reports of tumor seeding in those with distal CCA. In theory, the puncture route for a distal biliary lesion is usually nontransperitoneal and the needle tract is removed during the Whipple resection. Furthermore, a retrospective study found a significantly lower incidence of peritoneal carcinomatosis in patients with pancreatic adenocarcinoma after EUS-FNA as compared to percutaneous procedures (2.2% in the EUS group vs. 16.3% in the percutaneous group) [85].

In conclusion, EUS-FNA performs well in the detection and staging process of ECCA, although the relatively low negative predictive value is a fact that must be taken into consideration. Still, its nearly 100% positive predictive value is the utmost power when it comes to indicating aggressive treatment including surgery. Due to the concern of tumor seeding, the EUS-FNA of proximal lesions should be indicated with caution [86].

### 8.4. Intraductal Sonography (IDUS)

IDUS uses high-frequency (12-20 MHz) catheter-based probes of 2 mm diameter introduced into the biliary tract over a guidewire through the working channel of a duodenoscope. In most cases, preceding sphincterotomy is not necessary. IDUS is safe and technically easy to learn, but image interpretation remains challenging. It is used both for CCA detection and local staging. Lymph node assessment with this high-frequency ultrasound is not possible due to a penetration depth of only 20 mm.

IDUS distinguishes three layers of the normal bile duct wall: (1) inner hyperechoic corresponding to mucosa, (2) middle hypoechoic of smooth muscle fibers with fibroelastic tissue, and (3) outer hyperechoic corresponding to connective tissue.

Several diagnostic features of malignant strictures were described such as the disruption of the normal sonographic pattern, presence of a hypoechoic infiltrating lesion with irregular margins, and a tumor invasion into surrounding tissues. Malignancy is extremely likely if IDUS detects a tumor invasion into the hepatic artery, portal vein, or pancreatic parenchyma. Secondly, findings considered typical for a benign bile duct stricture include the preservation of the normal wall pattern, a homogeneous echo pattern, smooth margins, hyperechoic lesions, and absence of a mass lesion. The accuracy of IDUS in differentiating benign from malignant strictures ranges from 76 to 98% in the literature [87–89].

Other diagnostic features such as interrupted wall structure, the presence of a sessile tumor intraductally or outside the bile duct, or tumor size greater than 10 mm were suggested by Tamada et al. The likelihood of malignancy was as high as 97% when two or three of these features were present. To the contrary, an IDUS examination with no positive feature correlated with negative findings at the final diagnosis [90].

Even though IDUS has no capability for tissue sampling, it proved to be more accurate in distinguishing benign and malignant strictures than ERCP with transpapillary biopsy. In a retrospective study, IDUS was more specific (92% vs. 42%) and similarly sensitive (89% vs. 83%) as ERCP with biopsy. Compared to EUS, IDUS had higher sensitivity (91% vs. 75%) and specificity (80 vs. 75%) [91, 92].

IDUS has demonstrated to be a useful tool in predicting positive biopsy. When an intraductal sessile tumor could be visualized, the sensitivity of the biopsy was 92%. Moreover, as previously suggested, IDUS-directed bile duct sampling was found to be more sensitive than ERCP-guided sampling (87% vs. 67%) [28, 93].

As for the assessment of longitudinal tumor spread, IDUS proved better accuracy than ERCP (84% vs. 47%). In a study investigating bile duct wall thickness using IDUS in patients who had not undergone biliary drainage, in 95% of cases, the biliary wall of the common hepatic duct was not thicker than 1.8 mm unless the patients were diagnosed with primary sclerosing cholangitis or had longitudinal cancer extension along the bile duct [94]. A different study reported the mean length of longitudinal extension beyond the estimated measure by cholangiography to be about 6-10 mm for submucosal and 10-20 mm for mucosal spread [95].

The depth of tumor invasion can also be assessed by IDUS. When the outside hyperechoic layer is interrupted, a serosa invasion is suggested with an accuracy of 86%-93%. A vascular invasion is suspected if a high-echoic (“interface”) echo in between the tumor and vessel wall disappears. While diagnostic accuracy for portal vein and right hepatic artery invasion assessment was 86-100% and 92-100%, respectively, the visualization of the left and proper hepatic artery was poor. Therefore, CT and IDUS should be regarded as complementary staging methods [96–98].

### 8.5. Confocal Laser Endomicroscopy

Probe-based confocal laser microscopy is a method providing real-time, 400-fold-magnified imaging of the mucosa. The probe is introduced into the biliary tract through a catheter or the working channel of a cholangioscope. The intravenous injection of 10% fluorescein sodium is mandatory. A high diagnostic performance in malignant biliary strictures with accuracy of 79-82% has been reported. Nevertheless, this technology is costly and interpretation of images challenging [99–101]. Of note, in cases with prior biliary stenting, accuracy decreased to 45% in one study. Therefore, to decrease the rate of misclassification of benign stricture as malignant, usage of Paris (instead of Miami) Classification was recommended in this clinical scenario [102, 103].

## 9. Patients with PSC

The diagnosis of CCA in patients with primary sclerosing cholangitis (PSC) is cumbersome since background inflammation can obscure the clinical and morphological manifestation of the disease. According to the ESGE/EASL guidelines, ERCP with tissue sampling should be considered in patients with worsening symptoms, a rapid increase of cholestatic enzyme levels, or a new dominant stricture or progression of existing dominant strictures at MRCP. The diagnostic performance of DSOC was shown to be similar to non-PSC patients [104].

## 10. Conclusion

A rational utilization of cross-sectional imaging and endoscopic procedures in patients with clinical and laboratory suspicions of CCA is mandatory. The availability of advanced endoscopic methods, such as cholangioscopy and EUS, is increasing and makes the diagnostic algorithm easier. The limitation of tissue sampling resides in its low negative predictive value for malignancy. Despite the considerable progress in this field, further clinical research is needed.

## Abbreviations

CCA: Cholangiocarcinoma
ECCA: Extrahepatic cholangiocarcinoma
ICCA: Intrahepatic cholangiocarcinoma
PCCA: Proximal (perihilar) extrahepatic cholangiocarcinoma
DCCA: Distal extrahepatic cholangiocarcinoma
ERCP: Endoscopic retrograde cholangiopancreatography
EUS: Endoscopic ultrasound
EUS-FNA: Endoscopic ultrasound (-guided) fine needle aspiration
IDUS: Intraductal ultrasonography.
